# Beneficial Handling of Molecular Chaperones (Chaperonotherapy) in Glioblastoma and Neuroblastoma: Novel Therapeutic Targets or Potential Agents?

**DOI:** 10.3390/cells14181447

**Published:** 2025-09-16

**Authors:** Maria Antonella Augello, Nima Shadan, Giuseppa D’Amico, Rosario Barone, Celeste Caruso Bavisotto, Federica Scalia, Alessandra Maria Vitale

**Affiliations:** 1 Department of Biomedicine, Neuroscience and Advanced Diagnostics (BiND), University of Palermo, 90127 Palermo, Italy; mariaantonella.augello@unipa.it (M.A.A.); nima.shadan@unipa.it (N.S.); giuseppa.damico01@unipa.it (G.D.); rosario.barone@unipa.it (R.B.); celeste.carusobavisotto@unipa.it (C.C.B.); alessandramaria.vitale@unipa.it (A.M.V.); 2 Department of Medicine and Surgery, Kore University of Enna, 94100 Enna, Italy

**Keywords:** molecular chaperones, Heat Shock Proteins, Chaperone System, chaperonopathies, chaperonotherapies, nervous system tumors, glioblastoma, neuroblastoma, immune system, immunotherapy

## Abstract

Molecular chaperones, especially Heat Shock Proteins (HSPs), play complex, context-dependent roles in cancer, particularly in nervous system (NS) tumors like glioblastoma (GBM) and neuroblastoma (NB). They are often upregulated, promoting tumor growth, poor prognosis, and resistance to therapy and immune responses. This supports the potential of negative chaperonotherapy, aimed at inhibiting them. However, some studies suggest chaperones can also act as tumor suppressors in certain cancers, indicating that positive chaperonotherapy—enhancing or restoring their function—may be beneficial. For NS tumors, this latter area is still understudied. With emphasis on GBM and NB, in this review we address the potential of molecular chaperones, particularly HSPs, as therapeutic targets or agents. We discuss strategies to inhibit pro-tumorigenic chaperones as well as the underexplored potential of chaperone induction and immunomodulation. Ultimately, we examine the emerging use of pharmacological and chemical chaperones to improve treatment outcomes in these NS tumors. These strategies, whether applied alone or in combination, may offer significant benefits for GBM and NB, which are presently among the most aggressive and challenging tumors to manage.

## 1. Introduction

The Chaperone System (CS), made of molecular chaperones, chaperone co-factors, co-chaperones, and chaperone receptors and interactors, is a sophisticated network which regulates protein homeostasis (proteostasis) and quality control within the cell, assisting polypeptides folding at various stages, from nascent chains still bound to ribosomes, to fully folded proteins, and to aggregated or misfolded proteins targeted for degradation [1,2,3]. In addition, molecular chaperones also have other noncanonical functions that are not related to the maintenance of protein homeostasis [4]. For this reason, when abnormal in structure and/or function, they can become pathogenic and cause diseases, known as chaperonopathies, including cancer [5].

The main components of the CS are the Heat Shock Proteins (HSPs), which comprise multiple highly conserved families, categorized primarily according to their molecular weights. These proteins are constitutively expressed and induced under stressful conditions as a cellular defense mechanism to maintain proteostasis [3,6].

HSPs were found to be abnormally expressed in various tumor tissues compared to healthy ones, and seem to play a dual role, acting both as pro-tumorigenic and anti-tumorigenic factors, being useful as diagnostic and prognostic biomarkers, as well as promising therapeutic targets or agents [7]. In the latter case, pro-tumorigenic molecular chaperones, usually overexpressed in cancers and positively associated with cancer initiation and progression, can be targeted through the so-called “negative chaperonotherapy”, aiming to eliminate/block/inhibit them. Conversely, anti-tumorigenic molecular chaperones, usually downregulated in cancers, can be enhanced/induced through the “positive chaperonotherapy” [8]. Moreover, a promising novel anti-cancer strategy is represented by immunotherapy, aiming to “educate” the organism’s immune system to fight cancer. In this regard, molecular chaperones themselves, especially membrane-bound or extracellular chaperones, can be used as immunomodulators to improve the immune response against cancer cells, or as carriers of poorly immunogenic tumor peptides, to amplify antitumor immune response [9].

Canonical treatment for nervous system (NS) tumors, such as glioblastoma (GBM) and neuroblastoma (NB) includes surgical resection followed by radiotherapy, or a combination. However, even with this multimodal treatment strategy, patients continue to experience limited clinical benefits, as evidenced by low progression-free survival (PFS) and overall survival (OS) [10]. For instance, recent research indicates that the median survival of GBM patients ranges from only 9 to 19 months. This variation is influenced by several factors, including the patient’s age, the extent of surgical resection, and the tumor’s molecular characteristics [11,12].

For this reason, in recent years, numerous efforts have been made to identify more targeted therapies that can reduce common side effects of classical interventions, ameliorate patients’ quality of life, and prolong their survival [13,14,15,16]. In this regard, molecular chaperones, including HSPs, appear to be good candidates for anti-NS tumor interventions.

They were found to be highly expressed in various NS tumors, including GBM and NB, and play critical roles in their pathogenesis, progression, and therapeutic resistance to conventional treatments, such as radio-chemotherapies [17]. High-grade gliomas, medulloblastomas, and neuroblastomas inhabit a stressful intracranial milieu characterized by hypoxia, acidosis, and intense oxidative load. Rather than succumbing, these tumors co-opt the CS, most conspicuously the HSPs families, to stabilize oncoproteins, avert proteotoxic collapse and survive cytotoxic therapy [18,19]. Therefore, these HSPs might be targeted through the negative chaperonotherapy [20,21].

On the other hand, the same proteins that fortify malignant cells adopt an opposite behavior once they appear extracellularly, since they can “help” in targeting cancer cells. In this case, therapeutic interventions could rely on positive chaperonotherapy, or HSPs-based immunotherapy. For instance, glioma and medulloblastoma cultures constitutively expose the 70-kDa HSP (HSP70), the Glucose-regulating protein 78 (GRP78) and partner chaperones on the plasma membrane and secrete them within exosomes [18]. Exposed HSP70 functions as a danger signal binding to CD94/NKG2 and pattern-recognition receptors primes natural killer (NK) and dendritic cells. Clinical correlative studies extend pre-clinical observation: glioblastoma patients with elevated pre-treatment frequencies of CD56+/CD94+/CD69+ NK cells, which recognize membrane-HSP70, show significantly longer overall survival [22].

In this review, we discuss the potential of molecular chaperones, especially HSPs, as drug targets or agents against NS tumorigenesis, focusing on GBM and NB. We offer an overview of the therapeutic strategies targeting pro-tumorigenic HSPs and, on the other hand, those exploiting the immunomodulatory activity of some of them in both GBM and NB, that, to date, represents two of the most aggressive and challenging malignancies to treat, limiting the efficacy of currently used approaches.

## 2. Negative Chaperonotherapies Against GBM and NB

In various cancer, abnormally expressed molecular chaperones, especially HSPs, play a key pro-tumoral role regulating the main hallmarks of cancer, supporting the activation or inhibition of different signaling pathways such as sustaining cancer cells proliferation, inhibition of cell death, evading immune system response, inducing angiogenesis, activating cancer cells invasion and metastasis [7,23]. Therefore, targeting these “guilty” molecular chaperones (negative chaperonotherapy) offers promising innovative therapeutic strategies for cancer therapy or to enhance the effects of traditional anti-cancer treatments [24,25].

Regarding NS tumors, it has been observed that different HSPs are constitutively expressed or overexpressed [26,27,28,29], and often positively correlate with tumor progression and poor prognosis [27,28,30].

Below are listed some therapeutic strategies developed to inhibit pro-tumoral HSPs in the neuro-oncology field, especially against GBM and NB (Table 1).

### 2.1. 90-kDa Heat Shock Protein Inhibition in GBM and NB

The 90-kDa heat shock protein (HSP90)’s client proteins include various molecules associated with cancer development. Therefore, several hallmarks of cancer could be affected simultaneously when HSP90 function is lost or inhibited [80,81,82,83].

Benzoquinone ansamycins were the first class of natural HSP90 inhibitors to be discovered. Among these, geldanamycin (GA) is a naturally occurring antibiotic originally isolated from Streptomyces hygroscopicus in the 1970s. It bindfs with high specificity to the N-terminal ATP binding pocket of HSP90, blocking the chaperone in its ADP-bound conformation and preventing interactions with its client proteins, which results in their ubiquitination and proteasomal degradation [84,85]. Among them are numerous regulators of cell cycle progression. It has been demonstrated that GA treatment reduced the Cdc2-HSP90 and Cdc2-cyclin B1 complexes in a GBM cell line, inducing the ubiquitination and proteasomal degradation of Cdc2 and cyclin B1, ultimately leading to G2/M cell cycle arrest and reduced cell proliferation [31]. Similarly, the use of GA disrupted the interaction between HSP90 and its substrates EF-2 kinase and HIF-1α in different glioma cell lines, resulting in reduced cells’ clonogenicity and migration ability, and inhibiting the growth of glioma xenografts in nude mice [32,33].

Although it has potent anti-cancer activities in preclinical in vitro and in vivo studies, GA is not currently used in clinical practice due to its high hepatotoxicity [86]. Therefore, numerous less toxic and more water-soluble derivatives were developed, such as 17-allylamino-17-demethoxygeldanamycin (17-AAG) [87].

Several studies have investigated 17-AAG as a therapeutic agent alone or in combination with standard therapies against GBM, one of the most aggressive brain tumors in adults. Likely to GA, 17-AAG was able to arrest the growth and induce apoptosis in a large panel of genetically diverse human glioma cell lines by promoting downregulation or degradation of some HSP90′s client proteins [32,34,35,37]. The same effects were observed in orthotopic glioma mouse models, where the tumor growth was significantly delayed in animals receiving the 17-AAG compared to controls [32,34,35]. Moreover, 17-AAG acted as a radiosensitizer both in vitro and in vivo, inhibiting tumor growth in a significant manner compared to radiation alone [35,38], and synergized with the chemotherapeutic agent temozolomide (TMZ), enhancing its cytotoxicity [39]. The antiproliferative and cytotoxic effects of 17-AAG against GBM cell lines were significantly potentiated by the simultaneous silencing of HSP27, suggesting synergistic antitumor benefits of concomitant HSP27 knockdown and HSP90 inhibition [36].

Similarly to 17AAG, numerous other HSP90 inhibitors were able to suppress GBM growth and progression either in vitro, by inhibiting several key oncogenes and regulators of GBM biology, which are direct HSP90 client proteins, or in vivo, by inducing tumor regression in GBM-bearing mice and increasing animal survival [42,43,44,45]. Moreover, like 17AAG, some of these HSP90 inhibitors enhanced chemo and radio-sensitization in glioma cells by impairing DNA damage repair, and induced G2/M arrest and mitotic catastrophe as well as cell death by apoptosis/necrosis [43,46,47,48]. Likely, in vivo, these treatments enhanced radiosensitivity, inducing tumor regression and extending the median survival of tumor-bearing mice [43,47,48].

Therefore, HSP90 inhibitors like 17-AAG may have therapeutic potential in GBM, either as a single therapeutic strategy or in combination with other treatments [36,88] and should be considered for patients whose tumors remain refractory to most current treatments.

A NS tumor for which HSP90 inhibition as a therapeutic strategy has been less explored is NB. Classical HSP90 inhibitors, i.e., GA-derivatives 17-AAG or EC5, were reported to significantly block human NB growth and induce apoptosis in vivo, by decreasing Raf-1 and increasing the expression of cleaved PARP [40]. 17-AAG treatment was found to reduce cellular proliferation, viability, and migration/invasion, and induce cell death in two different NB cell lines by interfering with different HSP90-dependent molecular pathways involved in cancer and inducing the downregulation of numerous tumorigenic proteins [41].

Recently, the anticancer activity of the next-generation inhibitors XL-888 and Debio0932 has been investigated in vitro in a NB cell line. The obtained results showed that HSP90 inhibition affected different cancer-related processes, including tumor growth, cell proliferation, migration, invasion, metastasis, angiogenesis and apoptosis, thereby representing a good candidate for the development of novel therapeutic strategies against NB [49]. In another recent paper, HSP90 was found to be the target of a natural compound, i.e., epoxyazadiradione (EAD), a limonoid derived from Azadirachta indica, which was shown to exert an anti-cancer activity against NB. In particular, it was found that EAD inhibited the expression of Enolase1 and HSP90 in a NB cell line, resulting in reduced cell proliferation, enhanced apoptosis, and arrested cell-cycle progression at the SubG_0_ and G_2_/M phase [50].

Therefore, HSP90 inhibition appears as a promising therapeutic intervention in treating NB as well. Nevertheless, additional in vivo studies should be carried out to investigate its safety and tolerability.

### 2.2. 70-kDa Heat Shock Proteins Inhibition in GBM and NB

The 70-kDa heat shock protein (HSP70) family’s members, also known as the DnaK family HSPs, are localized in various subcellular compartments, as well as on the cell plasma membrane.

For instance, the heat shock protein A5 (HSPA5), also known as GRP78 or Bip, is a member of the HSP70 family that regulates endoplasmic reticulum (ER) stress and the activation of the unfolded protein response (UPR) cascades and helps in maintaining cell homeostasis following stresses [89]. HSPA5 expression was found significantly elevated in recurrent GBM specimens following treatment with chemotherapeutics and radiation compared with normal brain tissue, suggesting a potential link between increased protein expression and both tumor recurrence and resistance to chemo-radiotherapy [51,90]. Accordingly, HSPA5 downregulation or silencing increased the sensitivity of glioma cells to various chemotherapeutic agents, such as TMZ, 5-fluorouracil, irinotecan, etoposide, and cisplatin [51,52]. Other preclinical studies have investigated the ability of HSPA5 inhibitors, antibodies, and other agents that bind to and inhibit HSPA5 to reduce glioma cells’ viability and enhance the sensitivity to chemo-radiotherapy. For instance, the treatment of primary human GBM cells with OSU-03012 suppressed HSPA5 expression and resulted in cell death and enhanced radiosensitivity. Moreover, in vivo, the agent significantly suppressed tumor growth, an effect that was further enhanced by radiation exposure, and prolonged the survival of GBM-bearing mice, suggesting its use in combination with radiotherapy to treat GBM patients [53].

An antibody targeting the C-terminus of HSPA5 has been shown to suppress the proliferation of GBM cells and induce apoptosis by inhibiting the PI3K/AKT/mTOR signaling pathway in vitro, and to delay tumor growth in a heterotopic tumor mouse model when combined with ionizing radiation [54]. Recently, Zhu and colleagues have built a nanoplatform able to deliver an HSPA5 inhibitor (pifithrin-μ) and a radiosensitizer (gold nanosphere), providing synergistic radiotherapy and photothermal therapy. Preclinical studies demonstrated that this nanoplatform was able to activate pro-apoptotic UPR cascades, inhibiting tumor progression in human SW1783 GBM-bearing mice [55].

Behind HSPA5/GRP78/BiP, other members of the HSP70 family have been proposed as targets in GBM treatment. For instance, the synthetic cannabinoid agonist WIN55-212-2 exerted cytotoxic effects in vitro against two human GBM cell lines (GAMG and U251), inhibiting their migration, invasion capability, and clonogenicity [56]. The antitumor effects of WIN55-212-2 relied on the inhibition of HSP70, and the increased expression of p53 and cathepsin D. Moreover, the compound inhibited tumor growth and can be safely administered parenterally in vivo, suggesting it as an interesting candidate as a chemotherapeutic agent [56]. Another HSP70 inhibitor, AEC, an N-aminoethylamino derivative of colchicine, was found to penetrate C6 rat GBM and B16 mouse melanoma cells, inhibiting the HSP70 substrate-binding and refolding activities [57]. Moreover, it sensitized GBM cells to doxorubicin, inducing cell death [57].

HSP70 was found expressed on the plasma membrane of different cancer cells, including primary GBM and other NS tumors [58,91], but not on the corresponding normal cells, and plays a key role in the migration and invasiveness of brain tumor cells [58]. This observation opens the possibility to target these cells through aptamers, peptides, anticalins, and antibodies able to interact with the membrane-bound HSP70 [92,93,94]. For instance, recently Shevtsov and colleagues reported that the use of small HSP70 inhibitors (pifithrin-μ/PES and JG98) substantially reduced the invasiveness of cancer cells and significantly delayed brain tumor progression, ultimately increasing overall survival [58]. A noteworthy emerging process involves HSP70 and the initiation of cuproptosis, a newly identified type of programmed cell death that differs from ferroptosis and apoptosis, mainly triggered by the buildup of copper in cells [95]. Essential regulators of cuproptosis consist of FDX1, DLAT, LIAS, proteolipid acylation, pyruvate, α-ketoglutarate, and HSP70 [95,96,97]. Under dysregulation of copper homeostasis, HSP70 identifies and attaches to incorrectly folded or aggregated proteins, helping them fold properly or directing them to the proteasome for breakdown. This reduces the harmful impacts of protein toxicity in cells, but it activates an intense stress response to protein toxicity, mitochondrial dysfunction, and may lead to cuproptosis. Recent research has demonstrated the prognostic value of cuproptosis-related gene expression in GBM patients [98,99], suggesting that this mechanism and HSP70 could serve as both prognostic and therapeutic markers. Nonetheless, its function and influence are yet to be clarified; however, in oral squamous carcinoma the binding between the product of the clock gene *PER2* and the HSP70 has been related to induction of cuproptosis mediating the ubiquitinated degradation of AKT [100].

To date, the inhibition of Hsp70 family members as anti-cancer strategy against NB has been less investigated. Triptolide, a diterpene triepoxide extract from a Chinese plant, was found to decrease NB cells’ viability and induce apoptosis, and to reduce tumor growth in a NB orthotopic mouse model by inhibiting HSP70 expression, suggesting it as a promising novel therapy for NB, either alone or as part of a multidrug treatment, especially for patients with advanced-stage disease and high resistance to traditional chemotherapies [59,60]. Certainly, other studies clarifying the exact role of HSP70 family members in NB biology could promote the development of novel targeted therapies.

### 2.3. 60-kDa Heat Shock Proteins Inhibition in GBM and NB2.1

The 60-kDa heat shock protein (HSP60) family comprises various members collectively known as chaperonins. Among them is HSP60, a mitochondrial chaperonin that is one of the most multifaceted HSPs studied in the context of carcinogenesis, both as a valuable diagnostic and prognostic biomarker and a promising agent/target for treatment [101,102,103]. To date, most reports suggest HSP60 as a pro-tumorigenic factor, since its altered expression, localization, and some post-translational modifications positively correlate with tumor development and progression, as well as anti-cancer drug resistance and tumor recurrence [104,105,106,107]. Therefore, it appears as an attractive target for anti-cancer therapies [104,108].

The role of HSP60 in NS tumors has been less investigated. The protein was found to be considerably upregulated in GBM samples [19,109]. It was suggested that, similarly to other cancer types, it may have a cytoprotective role centered on survivin stabilization and the formation of the HSP60-p53 complex, which restrains p53 pro-apoptotic function [109]. Conversely, acute ablation of HSP60 by small interfering RNA may result in loss of the survivin mitochondrial pool and a parallel disruption of the HSP60-p53 complex, resulting in the activation of p53-dependent apoptosis [104,109].

The synthetic small-molecule KHS101 is known to target the chaperonin HSP60, which plays a key role in the maintenance of mitochondrial proteostasis. Accordingly, it has been shown that KHS101 selectively disrupted energy metabolism and induced autophagy-mediated cell death in various patient-derived GBM cell lines, regardless of their molecular subtypes, by targeting HSP60 chaperone activity. Moreover, it reduced tumor growth and increased survival of xenograft tumor-bearing mice [63]. Similarly, HSP60 knockdown in various stable GBM cell lines led to epithelial–mesenchymal transition (EMT), enhanced ROS generation through the disruption of mitochondrial respiratory chain complex I, and significantly suppressed cell growth and proliferation by activating the ROS-AMPK-mTOR pathway [64].

HSP60 was classically considered an intracellular protein, but in the last few years, evidence has shown that it can be released extracellularly, where it acts as a ligand for TLR-4 receptor [110]. Recently, curcumin has been shown to exert an anti-glioma effect in vitro, by inhibiting the inflammatory HSP60/TLR-4 signaling pathway [65]. Glioma cells treated with curcumin reduced the expression of HSP60 and likely its release and interaction with TLR-4, hindering the activation of the downstream pro-inflammatory pathway, mediated by MYD88 and NF-κB. Moreover, the treatment increased the expression of the apoptosis-related proteins caspase-3 and tumor suppressor gene p53 [65].

Similarly, it has been reported that curcumin can affect HSP60 expression, localization, and post-translational modifications in a NB cell line as well. In particular, the compound reduced the HSP60 cytoplasmic level, likely activating multiple intracellular molecular pathways that ultimately promote cell death [66]. Also in this case, one of the downstream targets of this curcumin-mediated HSP60 downregulation could be survivin, which is stabilized by mitochondrial HSP60 and promotes human NB cell lines’ survival [111].

Other members of the 60-kDa chaperonin family are those belonging to the eukaryotic cytoplasmic chaperonin CCT (chaperonin containing the T-complex polypeptide 1 [TCP1]), also known as TRiC (tailless complex polypeptide 1 ring) [112]. Some of these members were found overexpressed in glioma samples and cell lines, suggesting them as promising therapeutic targets [67,68]. For instance, the CCT8 was found to be significantly increased in gliomas and positively correlated with tumor grade and reduced survival [67]. Conversely, CCT8 siRNA-mediated knockdown in GBM cell lines arrested the cell cycle at the G1/S checkpoint, reduced cell proliferation, and suppressed the migration and invasion abilities of tumor cells following cytoskeleton dysregulation [67].

Similarly, CCT2 was found to be highly expressed in gliomas compared to normal brain tissues and negatively correlated with patients’ survival [68]. Studies in vitro and in vivo demonstrated that CCT2 promoted GBM progression by directly binding to KRAS, resulting in increased protein stability and upregulation of its downstream signaling [68]. Consequently, CCT2 inhibition through dihydroartemisinin, a derivative of artemisinin, resulted in the inhibition of the KRAS signaling pathway and suppressed GBM malignant progression, providing a new idea for GBM treatment [68]. CCT2 overexpression was also detected in NB tissues from pediatric patients and NB cell lines [20]. Exogenous stable expression of CCT2 enhanced GBM cells’ migration by remodeling the cell cytoskeleton. On the contrary, CCT2 depletion through a novel peptide CCT inhibitor CT20p decreased both cell viability and migratory ability, suggesting CCT2 as a novel therapeutic target for treating NB [20].

All eight subunits of the TRiC were found to be more abundant in EVs from neurosurgical aspirates of GBM patients compared to EVs from neurosurgical aspirates of low-grade glioma patients [113]. In particular, CCT6A had the greatest induction of expression and amplification in GBM and was significantly associated with reduced patient overall survival, suggesting it as a potential prognostic biomarker [113]. Moreover, it has been reported that CCT6A silencing reduced GBM cells’ invasion and migration, suggesting it as a promising therapeutic target as well [69].

Wang and colleagues found that the Y-box-binding protein 1 (YB-1) was strongly overexpressed in GBM tissues and cell lines, where it activated both mTORC1 and mTORC2 signaling pathways by increasing CCT4 production, which, in turn, promoted the mLST8 (MTOR-associated protein, LST8 homolog) folding and stability [114]. They also observed that the YB-1/CCT4/mLST8/mTOR axis promoted GBM growth in vivo in a mouse xenograft model, whereas the administration of RNA decoys specifically targeting YB-1 repressed tumor growth and increased survival [114].

### 2.4. 40-kDa Heat Shock Proteins Inhibition in GBM

The 40 kDa heat shock protein (HSP40) family, also referred to as the DnaJ family, comprises a large group of co-chaperones that function in concert with HSP70 proteins in promoting proteins folding and preventing aggregation. These proteins are collectively termed J proteins because they all contain a highly conserved J-domain, originally identified in the bacterial co-chaperone DnaJ of Escherichia coli. The role of its members in NS tumors is still under-investigated, and few studies have attempted to clarify the effects of their inhibition, and thus their potential as candidates for therapeutic interventions in NS tumorigenesis.

No studies have investigated the inhibition of HSP40 family members in NB. Regarding GBM, HSP47 was found to be significantly overexpressed in primary GBM cells and vessels, and was associated with tumor grade [70,71,115]. In particular, HSP47 stable overexpression in primary GBM cells promoted cell invasion, angiogenesis, and stem-like properties via the TGF-β pathway [115]. On the contrary, HSP47 knockdown by siRNAs significantly decreased cell viability, growth, migration, and invasion in vitro, and reduced tumor volume and vasculature in vivo [70,71]. Hopefully, future preclinical studies will elucidate the therapeutic efficacy of HSP40 inhibitors in neuro-oncology.

### 2.5. Small Heat Shock Proteins Inhibition in GBM

Different members of the small HSP family were found overexpressed in various NS tumors and correlated with tumor progression and poor prognosis [27,116]. HSP27 was found to be highly expressed in GBM cells [117]. This molecular chaperone is known for inhibiting apoptotic signaling pathways, such as caspase activation, promoting cancer cells’ proliferation and survival, and chemotherapy resistance [118]. Therefore, HSP27 inhibition represents an attractive anti-cancer strategy, especially to reduce the development of drug resistance [119].

Numerous naturally derived compounds with antioxidant properties have been studied in cancer research as potential therapeutic agents. Some of them have been shown to target HSP27. For instance, quercetin (3,3′,4′,5-7-pentahydroxyflavone), a natural flavonoid, in combination with TMZ efficiently suppressed human GBM cell survival in vitro and induced apoptosis through the inhibition of both HSP27 and HSP72, the activation of caspase 3 and 9, the release of cytochrome c from mitochondria, and a decrease in the mitochondrial membrane potential [61,72]. Moreover, silencing both HSPs using specific siRNAs made GBM cells vulnerable to apoptosis induction upon TMZ and quercetin treatments [62].

The treatment of GBM cell lines with the soluble epoxide hydrolase inhibitor t-AUCB induced the activation of p38 MAPK, MAPKAPK2, and HSP27 in GBM cell lines, resulting in cells protection from apoptosis [74]. Conversely, combining t-AUCB treatment together with the inhibitor of HSP27 phosphorylation, KRIBB, potentiated its cytotoxic effect and induced cell apoptosis [74]. The same authors also observed that quercetin treatment strengthened the cytotoxic and pro-apoptotic effect of t-AUCB against GBM cells, by inhibiting HSP27 and suppressing Atg7 expression [73]. Another natural compound, i.e., rosmarinic acid (RA), has been found to silence HSP27 and induce apoptosis by activating the caspase-3 pathway [75]. Moreover, apoptosis was induced at a higher level following HSP27 silencing and subsequent quercetin or RA treatment [75]. Similarly, siRNA-mediated-HSP27 silencing enhanced the therapeutic effect of resveratrol, a natural polyphenol with potent anti-cancer activity in a GBM cell line [21,76]. The combined treatment promoted cancer cells’ apoptosis by suppressing HSP27 expression and inducing caspase-3 activity. Moreover, the cytotoxic effect was more effective than the treatment with resveratrol alone and also compared to quercetin treatment [76].

In a similar manner, siRNA-mediated HSP27 knockdown sensitized GBM cells to subtherapeutic concentrations of 17-AAG and staurosporine, resulting in a higher reduction of cell viability and proliferation, as well as an increase in cell apoptosis [36].

Recently, it has been reported that HSP27 overexpression protected GBM cells from ferroptosis, a new type of non-apoptotic regulated cell death [120], following its phosphorylation. On the contrary, HSP27 inhibition promoted Fe2+ absorption and ROS production, ultimately leading to tumor cell death by ferroptosis [77,78].

Another member of the small HSP family, αB-Crystallin (αBc), was found overexpressed in highly migratory glioma cells and associated with a highly invasive phenotype. αBc siRNA-mediated depletion reduced glioma cells’ migratory ability and made them sensitive to various apoptotic inducers, suggesting it as an attractive therapeutic target [79].

Regarding NB, similarly to HSP70 and HSP40 families, the inhibition of the members of the small HSPs family has been relatively underexplored as an anti-cancer approach.

In summary, HSPs inhibition or silencing using of specific synthetic or natural inhibitors, targeted siRNAs, or various other agents, are emerging as promising therapeutic strategy for cancer treatment, especially regarding NS tumors, which are often difficult and challenging to treat and associate with poor prognosis, frequent recurrence, and development of resistance to conventional anti-cancer drugs [121]. Moreover, numerous findings have shown that the HSPs downregulation potentiates the effects of chemotherapeutics, radiotherapy, and other treatment modalities [39,52].

Interestingly, a greater effective treatment outcome was observed in malignant brain tumors treated with a combination of HSPs inhibitors. For instance, it has been recently observed that the siRNAs-mediated depletion of multiple HSPs (HSP27, HSP60, HSP70, or HSP90), made glioma cells more sensitive to apoptosis upon resveratrol treatment and decreased cell proliferation and colony-forming ability, by enhancing ROS generation, and inducing ER stress and UPR signaling [21]. Similarly, the inhibition of the Heat Shock Response, combined with bortezomib administration, promoted the death of glioma cells by inducing JNK phosphorylation and activation [122].

## 3. Positive Chaperonotherapies Against NS Tumors

Behind their role in supporting tumor growth and progression by modulating the main hallmarks of cancer and supporting drug resistance, molecular chaperones also exhibit onco-suppressive properties since they are involved in the body’s natural defense mechanisms against cancer [123,124]. Dysfunction of these “positive” chaperones results in the acquisition of a selective advantage by cancer cells, which acquire greater malignancy and resistance to therapies. Consequently, new strategies to fight cancer include exploiting the antitumor potential of such protective chaperones through their selective activation, enhancement, or restoration of function.

Evidence suggests that during the early stages of tumor development, chaperone-mediated autophagy (CMA) acts [125]. In general, CMA plays a key role in maintaining cellular homeostasis through a lysosome-dependent mechanism [126]. The CMA pathway selectively degrades some soluble cytosolic proteins containing the KFERQ-like motif, which is recognized by Hsc70. The formed protein complex is then targeted to the lysosomal membrane, where interaction with the cleavage variant A of lysosome-associated membrane protein-2 (LAMP-2A) results in its translocation and degradation inside the lysosome [127]. The molecular chaperone HSP90 also participates in the formation of the co-chaperone complex of CMA and has been seen to increase the stability and activity of LAMP-2A. Given its crucial role in slowing tumor growth, pharmacological modulation of CMA has emerged as a promising therapeutic strategy for both brain tumors and neurodegenerative disorders, where protein turnover dysregulation is a key pathological feature [125,128].

Several tumor suppressors are “client” proteins of molecular chaperones, which help them achieve a stable and functional conformation, thereby preventing misfolding, aggregation, and premature degradation [129]. For instance, the chaperonin TRiC is critical for the proper folding of the suppressor protein VHL (von Hippel-Lindau). After synthesis, VHL is initially stabilized by the chaperone HSP70, and subsequently, TRiC helps VHL to reach its native functional conformation for the formation of the VCB complex (VHL-Elongin B/C Complex). This complex prevents aberrant activation of pro-angiogenic genes under normoxic conditions through the degradation of the hypoxia-inducible factor HIF [130,131]. The misfolding of VHL protein may be particularly relevant in GBM as this tumor often exhibits hypoxia-driven progression, and a defective VHL-HIF axis could lead to angiogenesis, metabolic dysregulation, and therapeutic resistance [15,132]. Therefore, its appropriate folding is necessary to ensure its onco-suppressive activity.

Unfortunately, regarding NS tumors, the research on beneficial chaperones remains largely unexplored, as most studies over the years have focused on inhibiting chaperones that support cancer cell survival and resistance to chemotherapy and radiotherapy. Noteworthy, though, are HSP60′s anti-cancer activities mainly due to its involvement in the regulation of cell apoptosis in tumors not located in NS [101,109,133]. A 2016 study on hepatocellular carcinoma (HCC) found that, HSP60 levels are significantly reduced in HCC [133]; however, it has been demonstrated that high levels of HSP60 enhance cellular differentiation while suppressing invasion and metastasis, without influencing cell proliferation or death. Furthermore, HSP60 appears to inhibit tumors by promoting mitochondrial biogenesis [134]. Similarly, in clear cell Renal Cell Carcinoma (ccRCC), low HSP60 expression is associated with mitochondrial dysfunction, increased oxidative stress, enhanced glycolysis, and tumor progression via EMT. These observations suggest that restoring or upregulating HSP60 expression could represent a promising strategy to mitigate ccRCC and HCC progression by preserving mitochondrial function and limiting malignant transformation [135]. These studies provide a valuable basis for exploring the dual role of HSP60 in the biology of NS tumors as well.

In summary, enhancing the function of beneficial molecular chaperones could represent a key strategy in preventing NS cancer relapses, as these proteins help maintain proper protein folding and cellular homeostasis, thereby reducing the probability of molecular alterations that drive tumor transformation [136].

## 4. Immunotherapy Based on Molecular Chaperones

Immunotherapy is a promising new approach in the field of cancer therapy. It involves the natural use of the body’s immune system to recognize and attack tumor cells and has proven to be a valid alternative in treating recurrences, where surgery, chemotherapy, and radiotherapy are no longer possible [137].

As mentioned previously, different HSPs are highly expressed in various types of cancer and are associated with cancer progression and poor prognosis. Nevertheless, these upregulated HSPs, especially membrane-bound and extracellular ones, may be exploited as immunotherapeutic targets. In this regard, it has been shown that extracellular HSPs can bind to receptors on dendritic cells, promoting cross-presentation of their associated peptides and initiating and modulating immune responses [138,139]. Furthermore, HSPs can bind antigens present on tumor cells, and it has been seen, in vitro, that they can induce a specific vaccine response, making them the ideal candidates for immunotherapy [140]. In addition to interacting with dendritic cells, HSPs are also involved in the positive regulation of inflammatory cytokines, including TNF-α, IL-6, IL-10 and IL-12, nitric oxide (NO) and chemokines, promoting therefore the anti-tumor immune response in the host [141,142].

Regarding gliomas, immunotherapy is one of the most promising strategies [143,144]. It has been demonstrated that HSPs can be used to create tumor-specific vaccines, thereby inducing tumor rejection. Furthermore, the chaperones themselves have, as already mentioned, pro-inflammatory action, and can activate macrophages, dendritic cells, and microglia [145,146] (Table 2).

Heat shock protein (HSP) peptide complex 96 (HSPCC-96) is a personalized polyvalent vaccine consisting of antigenic peptides bound to the 96 kDa heat shock protein (HSP96) directly collected from surgically resected non-necrotic tumor mass, and able to trigger a specific antitumor response [147,148]. Numerous studies have demonstrated its safety and preliminary clinical efficacy in treating both recurrent and newly diagnosed GBM [149,150,151]. In 2013, for the first time, it was demonstrated the efficacy of HSPCC-96 vaccination that was able to induce a significant peripheral immune response in 11 out of 12 patients with recurrent GBM. In particular, the patients showed an increase in IFNγ production by NK cells and cytotoxic T lymphocytes, and a reduction in regulatory T lymphocytes, indicating an effective proinflammatory response against the tumor. Moreover, immune responders showed a longer survival compared to the single non-responder [149]. A subsequent phase II study was conducted on 41 patients with recurrent, surgically resectable GBM, again administering autologous HSPPC-96 vaccination following total tumor resection. Vaccinated patients showed a better overall survival rate compared to standards, and the efficacy and safety of the vaccine were also demonstrated, except in cases of patients with previous lymphopenia [150]. The same authors conducted a study to evaluate the benefit of autologous HSPPC-96 vaccination, in combination with radiotherapy and chemotherapy, in patients with newly diagnosed GBM [151]. Forty-six patients who met the inclusion criteria were enrolled, and survival was evaluated as the primary endpoint. Both median PFS and OS increased in patients subjected to surgical resection, followed by radiotherapy, oral TMZ administration, and concurrent vaccination. Moreover, better outcomes were found in patients having a lower expression of the programmed death ligand-1 (PD-L1) on myeloid cells’ surface, suggesting that the combination of PD-1/PD-L1 inhibition and vaccination could increase the efficacy of this immunotherapeutic approach, representing an effective alternative to the limited existing treatments [151]. Similarly, Ji and colleagues observed an increase in the PFS and median OS in newly diagnosed GBM patients vaccinated with HSPPC-96 in combination with the standard treatment, in addition to an increase in tumor-specific immune response (TSIR) [152]. To establish if the TSIR could be a good predictor of the vaccine efficacy, a subsequent retrospective study aimed to examine the T cell receptor (TCR) repertoires of tumor-infiltrating lymphocytes in long-term survivors (LTS) and short-term survivors (STS) among newly diagnosed GBM patients following HSPCC-96 vaccination. Using Next-Generation Sequencing (NGS) technology, the amount of T-cell receptor β (TCR-β) was determined. LTS exhibited a lower TCR repertoire diversity, and four TCR clones had a significantly higher abundance in LTS compared with STS. These results suggest that these TCR clones can predict clinical outcomes in response to HSPCC-96 vaccination and thereby be used to select GBM patients most likely to positively respond to it [153].

Superparamagnetic iron oxide nanoparticles (SPIONs) are an interesting and emerging category of nanovaccines that can activate dendritic cells (DCs). In a study, these nanoparticles were coated with recombinant HSP70 protein, known for its capacity to bind antigenic peptides. The obtained HSP70–SPIONs were able to bind tumor peptides and deliver them to DCs, stimulating a tumor-specific, CD8+ cytotoxic T cell response. C6 glioma-bearing rats vaccinated with DCs pulsed with HSP70-SPIONs and tumor lysates showed a delayed tumor progression and a better overall survival. Also in this case, it was observed a greater secretion of IFNγ in the serum and a greater infiltration of NK cells and CD8+ cytotoxic T lymphocytes [154]. The same authors observed that conjugating the HSP70-specific antibody (cmHSP70.1), which recognizes the membrane-bound HSP70, to the same superparamagnetic iron nanoparticles, they were retained in C6 GBM cells and glioma-bearing rats. The accumulation within HSP70-positive gliomas was further enhanced after a dose of ionizing radiation, suggesting the use of this system to detect tumor cells with MRI [155]. Moreover, when nanoparticles were functionalized with serine protease granzyme B, which specifically binds to membrane-bound HSP70, magnetic particles accumulated in the human U87 glioma cell line and induced apoptosis [156].

An experimental study demonstrated the therapeutic potential of the HSP70 protein in the treatment of GBM using an animal model, based on its known role in experimental anticancer vaccines. The key findings showed that local administration of HSP70 in rats bearing tumors inhibited tumor growth and significantly prolonged animal survival, particularly with repeated injections that led to the accumulation of HSP70 within tumor cells. This accumulation activated a robust antitumor immune response, positioning HSP70 as a promising immunomodulator. In particular, the treatment induced elevated production of interferon gamma (IFN-γ), enhanced activity of NK cells and CD8^+^ T lymphocytes and promoted tumor infiltration by immune cells [157].

The same authors tested this approach in pediatric patients with newly diagnosed malignant brain tumors who received intratumoral injection of recombinant HSP70 [158]. Following the treatment, a change in lymphocyte subpopulations was observed, with a shift from Th2 cell-derived cytokines toward Th1 cell-mediated immune response, a decrease in immunosuppressive T-regulatory cell level, and an increase in interleukin-10 level [158]. Further randomized clinical trials are necessary to stabilize the optimum dose of the chaperone, set the treatment schedule, and assess the clinical outcome.

Similarly to HSP70s, HSP47 has also been reported as a novel glioma-associated antigen for vaccination, since GBM patients with a positive cytotoxic T lymphocyte response to HSP47 showed a prolonged PFS and OS [159].

## 5. Pharmacological Chaperones and Chemical Chaperones

Another therapeutic strategy in the field of chaperonotherapies is the use of small organic compounds with chaperone-like activities, such as pharmacological chaperones and chemical chaperones [160,161,162,163].

A pharmacological chaperone is, by definition, a low-molecular-weight molecule of a chemical nature capable of binding a protein specifically and stabilizing it [164,165,166]. They are characterized by a specific and high-affinity interaction for the protein whose folding they must assist in vivo [167] and their mechanism of action is based on the binding of unstable folded (or partially folded) mutant proteins. Therefore, they cannot be used in cases where the protein is absent [164].

The working mechanisms of chemical chaperones, on the other hand, are not fully understood. It is reported that they stabilize misfolded proteins, prevent aggregation and incorrect interactions with other proteins and endogenous chaperones to allow their correct delivery and act especially when cells are stressed by heat and high salt concentrations [161,168,169,170,171].

Today, pharmacological chaperones and chemical chaperones are a promising therapeutic prospect for numerous pathologies [172].

Even concerning cancer, mutations in some genes, like those coding for p53, RB1, BRAF and NQO1, may compromise the ability of the protein to fold correctly and remain stable within the cell and in these cases, the use of natural and non-natural pharmacological chaperones has been proposed to restore the protein wild type state [173,174,175]. It has also been seen that some temperature-sensitive mutant forms of the p53 protein can be stabilized by chemical chaperones that help the folding and bring them back to their native conformation [176,177,178].

Although the use of pharmacological chaperones and chemical chaperones in the field of NS tumors is still little discussed, their use in the treatment of neurodegenerative diseases is widely established [179,180], thus opening a window on other frontiers, such as those covered by this review.

## 6. Concluding Remarks

Targeting molecular chaperones, especially HSPs, represents a promising strategy in cancer therapy, including NS tumorigenesis. Numerous in vitro, in vivo, and even preclinical studies have demonstrated that, depending on their context-specific roles, either the inhibition of pro-tumoral HSPs or the enhancement of anti-tumoral members can yield therapeutic benefits and potentiate the effects of more canonical anti-cancer therapeutic interventions like radio-chemotherapies, especially against those malignancies still challenging to treat like GBM and NB. In addition, various HSPs also possess an immunomodulatory activity, and this property has been exploited in immunotherapeutic approaches, including the development of HSP-based cancer vaccines and the use of HSP-peptide complexes to enhance tumor antigen presentation and the organism’s immune response. Future investigations should aim to integrate these multiple approaches to maximize the anti-cancer effect and minimize other off-target effects. For this purpose, the understanding of the dual role of molecular chaperones in cancer biology is necessary.

## Figures and Tables

**Table 1:** Summary of therapeutic agents used to suppress the pro-tumoral activity of molecular chaperones against GBMs and NBs.

Targeted Molecular Chaperones	Therapeutic Agent	Sample(s)	Effects	Reference(s)
HSP90	Geldanamycin	Glioma cell lines	Induced cell cycle arrest and reduced cell proliferation and migration by disrupting the interaction with the substrate proteins Cdc2, EF-2 and HIF-1alpha	[31,32,33]
	17-AAG	Glioma cell lines	Arrested cell growth and proliferation, and induced apoptosis by inhibiting some HSP90′s client proteins	[32,34,35,36,37]
		Orthotopic glioma mouse models	Inhibited tumor growth	[32,34,35]
		Glioma cell lines and glioma mouse models	Enhanced the effects of chemo-radiotherapy	[35,38,39]
		Human NB xenografts	Inhibited tumor growth and induced apoptosis by decreasing Raf-1 and increasing protein expression of cleaved PARP	[40]
		NB cell lines	Reduced cell proliferation, viability, and migration/invasion, and induced apoptosis by interfering with different HSP90-dependent molecular pathways	[41]
	NXD30001	GBM cells and GBM mouse models	Inhibited tumor growth by targeting the EGFR-PI3K-AKT axis and increased radiosensitivity	[42,43]
	YZ129 and its derivatives	GBM cells	Induced GBM cell-cycle arrest at the G2/M phase, promoted cells apoptosis and inhibited cells proliferation and migration by targeting the calcineurin-NFAT pathway and the PI3K/AKT/mTOR signaling axis	[44]
	AUY922	Immortalized and patient-derived GBM cell lines	Decreased cell viability and induced cell death by reducing some oncoproteins expression	[45]
	HSP990	GBM cell lines and GBM-bearing mice	Induced GBM cells cell-cycle arrest at the G2/M phase and death by suppressing the AKT signaling, and increased radiosensitivity	[46,47]
	NW457	GBM cell lines and the orthotopic GBM mouse model	Reduced tumor cell proliferation and tumor progression, and increased radiosensitivity	[48]
	Small interfering RNA	GBM cells	Made cells more sensitive to apoptosis upon resveratrol treatment by inducing ER stress and UPR cascades	[21]
	XL-888 and Debio0932	NB cell line	Affected different cancer-related processes, including tumor growth, cell proliferation, migration, invasion, metastasis, angiogenesis, and apoptosis	[49]
	EAD	NB cell line	Suppressed cell proliferation, enhanced the rate of apoptosis, and arrested cell cycle at the SubG_0_ and G_2_/M phases	[50]
	EC5	Human NB xenografts	Inhibited tumor growth and induced apoptosis by decreasing Raf-1 and increasing protein expression of cleaved PARP	[40]
HSPA5/GRP78/Bip (HSP70 family)	Small interfering RNA	GBM cell lines	Inhibited glioma cells’ growth and increased their sensitivity to various chemotherapeutics	[51,52]
	OSU-03012	GBM cells and GBM-bearing mice	Induced cell death, suppressed tumor growth, and enhanced radiation efficacy	[53]
	Anti-HSPA5 antibody	GBM cells and the heterotopic tumor mouse model	Suppressed cell proliferation and induced apoptosis by inhibiting the PI3K/AKT/mTOR signaling pathway, and delayed tumor growth in vivo in combination with radiation	[54]
	Pifithrin-μ	GBM-bearing mice	Inhibited tumor progression by activating pro-apoptotic UPR cascades	[55]
HSP70	WIN55-212-2	GBM cell lines	Inhibited cell migration, invasion, and clonogenicity	[56]
	AEC	GBM cells	Sensitized cells to doxorubicin, inducing cell death	[57]
	pifithrin-μ/PES and JG98	Immortalized glioma cell lines, neuro-oncologic patient-derived cells and orthotopic brain tumor model	Reduced cell migration and invasiveness, delayed brain tumor progression and increased overall survival	[58]
	Small interfering RNA	GBM cells	Made cells more sensitive to apoptosis upon resveratrol treatment by inducing ER stress and UPR cascades	[21]
	Triptolide	NB cells and NB orthotopic mouse model	Decreased cell viability and reduced tumor growth in vivo	[59,60]
HSP72 (HSP70 family)	Quercetin	GBM cells	Sensitized cells to temozolomide and promoted apoptosis by activating caspase 3 and 9, inducing cytochrome c release, and decreasing the mitochondrial membrane potential	[61]
	Small interfering RNA	Glioma cells	Sensitized cells to temozolomide and quercetin and induced apoptosis by decreasing mitochondrial membrane potential, increasing the release of cytochrome c, and activating caspase 3 and caspase 9	[62]
HSP60	Synthetic molecule KHS101	Patient-derived GBM cell lines and xenograft tumor-bearing mice	Disrupted mitochondrial-dependent energy metabolism, reduced tumor growth, and increased survival	[63]
	Anti-HSP60 shRNA	GBM cell line	Inhibited cell proliferation by activating the ROS-AMPK-mTOR pathway	[64]
	Small interfering RNA	GBM cells	Made cells more sensitive to apoptosis upon resveratrol treatment by inducing ER stress and UPR cascades	[21]
	Curcumin	Glioma cells	Blocked the inflammatory HSP60/TLR-4 signaling pathway and promoted tumor cell apoptosis	[65]
		NB cells	Promoted cancer cells death	[66]
CCT8	Small interfering RNA	GBM cell lines	Decreased cell proliferation, migration, and invasion capacity and made	[67]
CCT2	Dihydroartemisinin	GBM cell lines and GBM animal models	Inhibited the proliferative activity, invasion, and migration ability of GBM by targeting the CCT2-KRAS axis	[68]
	CT20p	NB cell lines	Reduced cell viability and migration	[20]
CCT6A	Small interfering RNA	GBM cell lines	Reduced cell migratory and invasive capacity	[69]
HSP47	Small interfering RNA	Glioma cells	Reduced cell viability, growth, migration, and invasion	[70,71]
	Small interfering RNA and anti-HSP47 shRNA	Glioma xenograft mouse model	Reduced tumor growth and angiogenesis	[70,71]
HSP27	Quercetin	GBM cells	Sensitized cells to temozolomide and promoted apoptosis by activating caspase 3 and 9, inducing cytochrome c release, and decreasing the mitochondrial membrane potential	[61,72]
			Induced cell death by blocking autophagy and strengthening the cytotoxic effect of the epoxide t-AUCB	[73]
	KRIBB3	GBM cells	Potentiated the cytotoxic effect of t-AUCB and induced cell apoptosis by increasing caspase-3 activity	[74]
	Small interfering RNA	Glioma cells	Sensitized cells to temozolomide, quercetin, resveratrol, rosmarinic acid, and staurosporine, and induced apoptosis	[21,36,62,75,76]
	Rosmarinic acid	Glioma cells	Induced cell death by activating caspase 3	[75]
	Anti-HSP27 sgRNA and shRNA	GBM cells	Promoted erastin-induced ferroptosis	[77,78]
αB-Crystallin	Small interfering RNA	GBM cells	Reduced the cells’ migratory ability and sensitized them to various apoptotic inducers	[79]

Abbreviations: 17-AAG, 17-allylamino-17-demethoxygeldanamycin; AKT, Protein kinase B; AMPK, AMP-activated protein kinase; CCT, Chaperonin-containing tailless complex polypeptide 1; GBM, glioblastoma; EAD, Epoxyazadiradione; EF, Elongation factor; ER, Endoplastic Reticulum; HIF, Hypoxia-inducible factor, KRAS, Kirsten rat sarcoma virus; NB, neuroblastoma; HSP, Heat Shock Proteins; mTOR, mammalian Target of Rapamycin; PARP, Poly (ADP-ribose) polymerase; PI3K, Phosphoinositide 3-kinases; Raf, Rapidly Accelerated Fibrosarcoma; shRNA, short harpin RNA; sgRNA, single guide RNA; TLR, Toll-like Receptor; UPR, Unfolded Protein Response.

**Table 2:** Immunotherapy strategies based on molecular chaperones.

Involved Molecular Chaperones	Therapeutic Agent and Mechanism of Action	Model(s)	Systemic Effect(s)	Reference(s)
HSP96	Autologous polyvalent vaccine, consisting of antigenic peptides bound to HSP96 (HSPCC-96) → increased IFNγ and cytotoxic T lymphocytes and reduced regulatory T lymphocytes	Phase I/II clinical trials in patients with newly diagnosed and recurrent GBM	Proinflammatory response against the tumor and improved PFS and OS	[147,148,149,150,151,152,153]
HSP70	HSP70 conjugated to superparamagnetic iron oxide nanoparticles (SPIONs) → stimulation of a tumor-specific CD8+ cytotoxic T cell response	C6 GBM cells/C6 glioma-bearing rats/U87 glioma cells	Proinflammatory response against the tumor, delayed tumor progression and increased OS	[154,155,156]
	Local administration of HSP70 → infiltration by immune cells	GBM bearing rats/Clinical trial in pediatric patients with newly diagnosed brain tumors	Antitumor immune response, tumor growth inhibition and prolonged survival	[157,158]
HSP47	Novel glioma-associated antigen → GBM patients with a positive CTL response to HSP47	Primary GBM tissues	Longer PFS and OS	[159]

Abbreviations: CTL, cytotoxic T lymphocyte; GBM, glioblastoma; IFNγ, interferon γ; PFS, progression-free survival; OS, overall survival. Arrows (→) indicate the mechanism of action.

## Data Availability

No new data were created or analyzed in this study.

## Abbreviations

The following abbreviations are used in this manuscript:

17-AAG	17-allylamino-17-demethoxygeldanamycin
αBc	αB-Crystallin
AKT	Protein kinase B
AMPK	AMP-activated protein kinase
ccRCC	clear cell Renal Cell Carcinoma
CCT	Chaperonin-containing tailless complex polypeptide 1
CMA	Chaperone-Mediated Autophagy
CS	Chaperone System
DC	Dendritic Cells
EAD	Epoxyazadiradione
EF-2	Elongation factor-2
EMT	Epithelial–Mesenchymal Transition
ER	Endoplasmic Reticulum
GA	Geldanamycin
GBM	Glioblastoma
GRP78	Glucose-regulating protein 78
HCC	Hepatocellular Carcinoma
HIF	Hypoxia-inducible factor
HSPs	Heat Shock Proteins
HSP90	90 KDa Heat Shock Proteins
HSP70	70 KDa Heat Shock Proteins
HSP60	60 KDa Heat Shock Proteins
HSP40	40 KDa Heat Shock Proteins
HSPA5	Heat Shock Protein A5
HSPCC-96	Heat shock protein peptide complex 96
IFN	Interferon
KRAS	Kirsten rat sarcoma virus
LAMP-2A	Variant A of lysosome-associated membrane protein-2
LTS	Long-term survivor
mTOR	mammalian Target of Rapamycin
NGS	Next-Generation Sequencing
NK	Natural Killer
NO	Nitric Oxide
NS	Nervous System
OS	Overall survival
PARP	Poly (ADP-ribose) polymerase
PD-L1	Programmed Death Ligand-1
PI3K	Phosphoinositide 3-kinases
PFS	Progression-free survival
Raf	Rapidly Accelerated Fibrosarcoma
shRNA	short harpin RNA
sgRNA	single guide RNA
STS	Short-term survivor
RA	Rosmarinic Acid
RNA	Ribonucleic acid
TCP1	Tailless complex polypeptide 1
TCR	T cell receptor
TLR	Toll-like Receptor
TMZ	Temozolomide
TRiC	Tailless Complex Polypeptide 1 Ring Complex
TSIR	Tumor-Specific Immune Response
UPR	Unfolded Protein Response
VHL	von Hippel-Lindau
YB-1	Y-box-binding protein 1
